# Blinding in tDCS Studies: Correct End-of-Study Guess Does Not Moderate the Effects on Associative and Working Memory

**DOI:** 10.3390/brainsci12010058

**Published:** 2021-12-31

**Authors:** Marija Stanković, Marko Živanović, Jovana Bjekić, Saša R. Filipović

**Affiliations:** 1 Human Neuroscience Group, Institute for Medical Research, University of Belgrade, 11000 Belgrade, Serbia; marija.stankovic@imi.bg.ac.rs (M.S.); sasa.filipovic@imi.bg.ac.rs (S.R.F.); 2 Institute of Psychology and Laboratory for Research of Individual Differences, Department of Psychology, Faculty of Philosophy, University of Belgrade, 11000 Belgrade, Serbia; marko.zivanovic@f.bg.ac.rs

**Keywords:** transcranial direct current stimulation (tDCS), blinding, placebo, sham, associative memory, working memory, end-of-study guess

## Abstract

Transcranial direct current stimulation (tDCS) has become a valuable tool in cognitive neuroscience research as it enables causal inferences about neural underpinnings of cognition. However, studies using tDCS to modulate cognitive functions often yield inconsistent findings. Hence, there is an increasing interest in factors that may moderate the effects, one of which is the participants’ beliefs of the tDCS condition (i.e., real or sham) they received. Namely, whether participants’ correct guessing of sham condition may lead to false-positive tDCS effects. In this study, we aimed to explore if participants’ beliefs about received stimulation type (i.e., the success of blinding) impacted their task performance in tDCS experiments on associative (AM) and working memory (WM). We analyzed data from four within-subject, sham-controlled tDCS memory experiments (*N* = 83) to check if the correct end-of-study guess of sham condition moderated tDCS effects. We found no evidence that sham guessing moderated post-tDCS memory performance in experiments in which tDCS effects were observed as well as in experiments that showed null effects of tDCS. The results suggest that the correct sham guessing (i.e., placebo-like effect) is unlikely to influence the results in tDCS memory experiments. We discuss the results in light of the growing debate about the relevance and effectiveness of blinding in brain stimulation research.

## 1. Introduction

Over the last ten years, non-invasive brain stimulation (NIBS) techniques have emerged as promising tools for cognitive enhancement [1,2,3]. One of the most widely used of these techniques is transcranial direct current stimulation (tDCS), which uses a weak electrical current that passes through the skull and the brain between electrodes of opposite charge positioned at the surface of the scalp [4,5]. The current flow causes the ionic movement across the neuronal membranes, which generates a change in membrane polarization and thus makes the neurons within the created electrical field more or less likely to spontaneously fire action potentials, depending on the polarity of the nearest electrode [5,6]. The growing popularity of tDCS in both the research and clinical setting stems from its safety, good tolerability, easy application, and affordability [7,8]. Furthermore, the relative ease of delivering sham (placebo) stimulation enables researchers to conduct basic cognitive neuroscience experiments and make causal inferences about neural foundations of processes they want to investigate [4,5]. This is why tDCS has been a widely used tool to modulate different cognitive functions in both healthy and clinical populations (for a comprehensive overview see recent reviews and meta-analyses [9,10,11,12,13]).

Even though neurophysiological and behavioral effects of tDCS have been demonstrated across a wide range of cognitive and motor functions [14,15,16,17,18], the findings were not always consistent, which raised skepticism about its effectiveness [19,20].

Nevertheless, the question we aim to tackle herein is not whether tDCS has or has no effects on cognition, but to better understand what might lead to tDCS effects which are sometimes hard to replicate. Some of the factors that were prominently pointed out as plausible reasons for the variability of the findings include differences in experimental designs, such as stimulation settings and parameters, as well as low statistical power due to small sample sizes [21,22,23]. To improve the quality and reproducibility of tDCS research, guidelines that would lead to more consistent results have been developed. They argue that one of the conditions necessary for adequate estimation of tDCS effects is successful blinding—making sure that the participants are unaware of the stimulation type they receive (i.e., real or sham) [8,24]. Namely, compromised blinding gives rise to the possibility that subjective beliefs of participants about the stimulation type they received influence the results, i.e., that the placebo-like effect in tDCS trials occurs.

To assess the effects of tDCS, a sham condition is introduced in the experimental designs as a control, i.e., a “neutral” point of reference to be used for comparison of the performance following active tDCS. Sham stimulation may include delivering a current of very low intensity during the whole simulation period: the current delivered produces cutaneous sensations but is considered not strong enough to pass through the scalp and have physiological effects [25]. However, in most of the studies, the sham stimulation protocols include brief ramp-up/ramp-down stimulation (30–60 s) to induce cutaneous sensations similar to the active stimulation (e.g., tingling or itching sensations around the location of electrodes), while no current is delivered throughout the rest of the session. The brief stimulations at the beginning and the end do not affect cortical excitability [26]. A body of research showed that participants could not tell a difference between this type of sham protocol and active stimulation (e.g., [27,28,29]). However, recently, the suitability of this protocol has been questioned. Several papers argued that the blinding in previous tDCS studies had been either compromised or inadequately assessed and reported [30,31,32,33,34,35].

Participants’ perceptions about the given intervention can lead to the occurrence of a placebo-like effect. There are two possible mechanisms through which tDCS can lead to a placebo effect. They include participants’ awareness of the stimulation condition they are receiving and their expectancy of tDCS effects. In tDCS experiments on cognition, if the participants are aware of the received stimulation type, they can engage more (during real stimulation) or less (during sham stimulation) while completing cognitive tasks. On the other hand, expectations may influence the outcomes if participants anticipate the improvement of their performance because of the assumed tDCS benefit [36]. Moreover, the interaction between awareness and expectations may further influence the results of tDCS experiments. In this context, the pertinent question is how participants’ subjective beliefs about the stimulation type they receive affect task performance, especially if blinding was unsuccessful.

There is evidence that tDCS may have a strong placebo effect that significantly influences task performance and that beliefs about receiving the real or sham tDCS could impact participants’ behavior [37,38,39]. One recent report even suggested that individuals’ opinions about having received the real stimulation better predicted the investigated outcome measure than the actual intervention [30]. However, there are still only a few studies that have addressed the impact of participants’ subjective beliefs on the effects of tDCS, especially when it comes to cognitive measures.

To the best of our knowledge, no studies have evaluated the possible placebo tDCS effects on associative memory (AM), and only one study has done so with working memory (WM) performance [38]. Hence, here, we explored whether participants’ beliefs about stimulation types they received had affected their performance across four tDCS experiments on memory. Namely, we re-analyzed the data from two studies on tDCS effects on AM [40] and WM [41]. Bjekić et al. [40] showed facilitatory effects of anodal tDCS of the posterior parietal cortex (PPC) on AM, while experiments reported by Živanović et al. [41] assessing the effects of tDCS over PPC and dorsolateral prefrontal cortex (DLPFC) on verbal and spatial working WM resulted in mixed findings. Since both studies applied a within-subjects design with the same type of sham-control protocol as well as end-of-study guess to evaluate blinding, in this paper, we reevaluated the reported effects in the context of the correct sham guessing.

## 2. Materials and Methods

### 2.1. Experimental Design

The data from four experiments were analyzed. All experiments employed a within-subject, crossover, sham-controlled design with a counterbalanced order of stimulation conditions and parallel forms of the memory tasks. The overview of the main aspects of methods is presented in Table 1, while a detailed methodology has been presented elsewhere [40,41].

**Figure 1:**
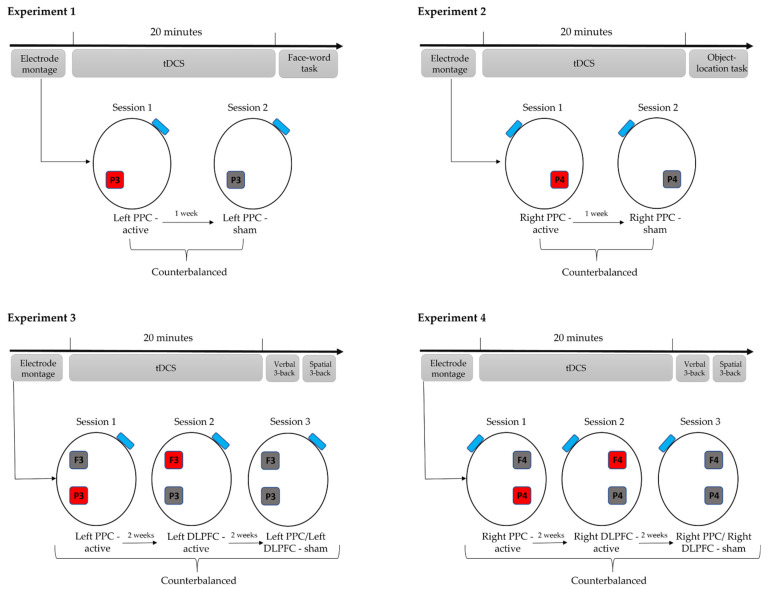
Experimental procedures for Experiments 1–4. The active electrode (anode, marked red) was placed over the cortical targets, i.e., left PPC (P3 site of the International 10–20 EEG system) in Experiment l, right PPC (P4 site of the International 10–20 EEG system) in Experiment 2, left PPC and left DLPFC (P3 and F3 site of the International 10–20 EEG system) in Experiment 3, and right PPC and right DLPFC (P4 and F4 site of the International 10–20 EEG system) in Experiment 4. In each experiment, the return electrode (cathode, marked blue) was placed over the contralateral cheek. The inactive electrode is marked grey. The participants performed memory tasks after the stimulation (offline protocol), and the order of the stimulation conditions was counterbalanced across all experiments. Note: tDCS—Transcranial Direct Current Stimulation; PPC—Posterior Parietal Cortex; DLPFC—Dorsolateral prefrontal cortex; EEG—Electroencephalography.

The experimental procedures are presented in Figure 1. Experiments 1 and 2 consisted of one anodal and one sham tDCS session. In Experiments 3 and 4, the participants went through three tDCS conditions, i.e., two anodal tDCS and one sham stimulation, each delivered in a separate session. The order of the conditions was counterbalanced; thus, participants were split into equal subgroups based on the arranged sequence of tDCS conditions they would receive. Namely, in Experiments 1 and 2, there were two subgroups (Sham—Real; Real—Sham), while in Experiments 3 and 4, three subgroups were formed (Real DLPFC—Real PPC—Sham; Real PPC—Sham—Real DLPFC; Sham—Real DLPFC—Real PPC). In each session of Experiments 3 and 4, three electrodes were placed on the participant’s head regardless of the active/sham stimulation locus to make it harder for them to guess the stimulation type they had received. To control for carryover effects in memory tasks, sessions were at least one week apart in Experiments 1 and 2, or two weeks apart in Experiments 3 and 4. During the stimulation, the participants completed diverse cognitive tests to raise cognitive engagement and attention, and after the stimulation, they performed the main memory tasks.

### 2.2. Participants

A total of 83 young, healthy participants took part in 4 memory experiments: 41 in Experiments 1 and 2 (20 and 21, respectively) and 42 in Experiments 3 and 4 (21 in each). All participants were right-handed, naïve to tDCS, had a normal or corrected-to-normal vision, and satisfied common inclusion criteria for tDCS studies [42].

### 2.3. Sham and Real tDCS

In the anodal tDCS condition, the current intensity was 1.5 mA (Experiments 1 and 2) and 1.8 mA (Experiments 3 and 4). The 5 cm × 5 cm rubber electrodes, in saline-soaked sponge pockets, were used in all experiments. The constant current was delivered for 20 min with a 30 s fade in/out period. In the sham condition, electrode positioning was kept the same, and the current was briefly delivered only at the beginning and at the end of the 20-min period. Namely, the current ramp-up was immediately followed by a ramp-down over the first and last 60 s of the protocol. The sham protocol in Experiments 3 and 4 was delivered via either the frontal (F3/F4) or posterior electrode (P3/P4), which was randomized across participants.

### 2.4. Memory Assessment

For the assessment of AM, a face–word memory task was used in Experiment 1. The task consisted of two learning and two test blocks. In the learning block, participants were successively presented with 20 face–word pairs and instructed to memorize as many as they could. Subsequently, the pictures of the same 20 faces were mixed with 30 lures (i.e., new faces) and participants were asked whether they recognize the face from the previous block and if so, to write in the word that face had been paired with. The participants had another chance to learn the same face–word pairs in the second learning block, which was, again, followed by the second test block with face-cued word recall. The outcome measure for this task was the number of correctly recalled face–word pairs after each test block.

In Experiment 2, AM was assessed with the object–location memory task, which also consisted of two learning and two test blocks. The stimuli were 15 colored pictures of common objects/living things (e.g., furniture, animals, etc.) that successively appeared across a 4 × 4 grid in the learning block. Each stimulus had its position in the grid, and the participants were instructed to memorize the locations of the given stimuli. In the test block, the same pictures were successively presented above the grid, and the participant’s task was to recall the position in the grid in which the stimuli previously appeared.

Verbal and spatial 3-back tasks were used for WM assessment (Experiments 3 and 4). Both tasks had one practice and five test blocks. In the verbal 3-back task, letters successively appeared on the screen. In the spatial 3-back task, the stimulus was a black square that successively changed its position in the 3 × 3 white grid. In both tasks, the participants were instructed to respond via keypress when the stimulus on the screen was the same as the one presented three trials before (25% of trials). WM scores were calculated as a percentage of hits for each 3-back task. Additionally, performance speed was evaluated by analyzing the reaction time (RT) for correctly identified targets, i.e., hits.

All memory tasks were programmed and administered in OpenSesame (OpenSesame Inc., Portland, OR, USA) [43] and the memory performance was automatically scored.

### 2.5. Blinding Assessment

To estimate whether blinding was successful, researchers typically adopt the end-of-study guess approach, when participants in both active and control groups are asked to guess if they have received the active or sham stimulation after the session [34] or, in crossover designs, to try to guess in which session they have received the sham stimulation [44]. Utilization of the end-of-study guess to assess the successfulness of blinding is a frequent approach in tDCS studies [28,32,33,45,46,47]. This approach was adopted to evaluate the successfulness of blinding across all experiments analyzed in this study.

Prior to the experiment, participants were informed that there will be a real and sham stimulation but were blinded for the type of stimulation they would receive in each session, i.e., they were not informed about the order in which they would receive them. At the end of the experiment (i.e., after the last experimental session), the participants were asked to guess the order of the tDCS conditions they had received. Specifically, they were asked to try to guess the session in which they received the sham stimulation. This means that in Experiments 1 and 2, the gap between guessing and the first session was 7 days, and in Experiments 3 and 4, it was 28–30 days for most participants. The participants noted their answers and were afterward told if they had guessed correctly or not.

### 2.6. Analytical Approach and Statistical Analysis

The data were analyzed in JASP (version 0.16.0.0 for Windows). First, we assessed whether blinding was successful in each experiment using the Chi-square test to estimate whether stimulation type guessing was above the chance level (50% in Experiments 1 and 2 and 33.3% in Experiments 3 and 4) and re-analyzed the dataset for tDCS effects on memory across all experiments.

In tDCS studies, the potential placebo-like effects from inadequate blinding are usually not directly observable from the data, but, if present, they are embedded in the interaction between tDCS conditions’ effects and the effects of the subjective beliefs regarding the type of stimulation received. Therefore, in a series of mixed two-way ANOVAs, we assessed the interaction between the tDCS condition (real vs. sham), used as a within-subject factor, and the subjective beliefs of stimulation type received (guessed sham correctly vs. incorrectly), as a between-subject factor.

**Figure 2:**
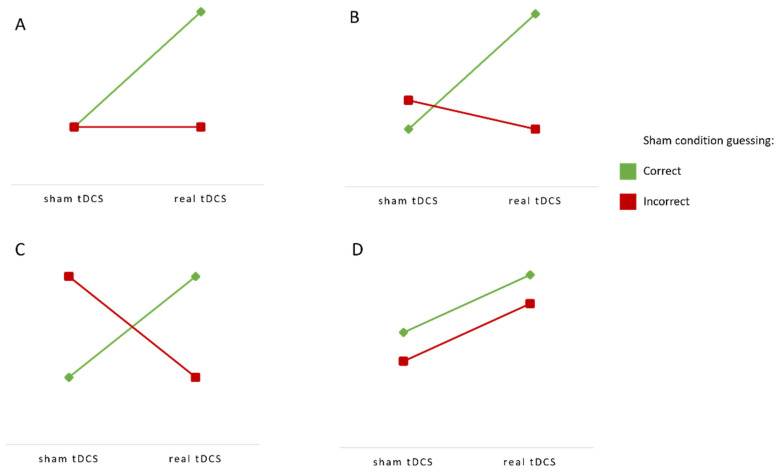
Interpretation of interaction effects between sham condition guessing and the actual tDCS condition. (**A**,**B**): Significant main effect of stimulation with significant interaction. (**C**): No main effect of stimulation with significant interaction. (**D**): Significant main effect of stimulation with non-significant interaction. (**A**–**C**) show that a correct sham guess moderates tDCS effects, while (**D**) shows that successful sham-guessing has no impact on the tDCS effects. Finally, the absence of both a stimulation and interaction effect would result in the same cognitive performance across all levels of both variables.

Since the interpretation of the interaction to assess the effect of a moderating variable is not always straightforward, here, we outline possible outcomes and their meaning (Figure 2). The significant interaction effect would suggest that correct and incorrect guess groups differ in terms of tDCS effects, which would be interpreted as the existence of a placebo-like effect. Thus, the significant interaction effect, in the case of significant tDCS effects, would suggest that the ones who accurately guessed the stimulation condition would score higher following real than following sham tDCS, while the participants that did not guess the sham condition would have similar performance in both conditions (Figure 2A) or even higher after sham tDCS due to false beliefs (Figure 2B). In the case of non-significant tDCS effects, the significant interaction effect would mean that the beliefs about the real vs. sham stimulation would account for all differences in the performance (Figure 2C). In contrast, the absence of the interaction effect between sham-guessing and the actual intervention would mean that the observed (facilitatory) effects of tDCS exist regardless of the subjective beliefs about the stimulation (Figure 2D).

In addition to the traditional frequentist significance tests, we adopted the Bayesian approach to provide more credible conclusions about both the existence and the absence of the effects [48]. In Bayesian inference, the Bayes factor (BF_10_) represents the odds of observing the data under the alternative hypothesis (H1) relative to observing it under the null hypothesis (H0). For theoretical background and more details on the Bayesian approach, see [49,50,51]. In this study, we assessed the strength of evidence towards H1/H0, where H1 is the model with an interaction effect between sham-guess and stimulation type on the memory performance, and H0 is the model with no interaction. BF_10_ for interaction is calculated as BF_10(main effects+interaction)_/BF_10(main effects)_. We interpreted Bayes factors according to the classification scheme adopted by JASP [52]. Namely, BF_10_ of 1–3 was interpreted as anecdotal, BF_10_ of 3–10 as moderate, and BF_10_ > 10 as strong evidence in favor of the H1 compared to the H0. BF_10_ = 1 is interpreted as equal probability of H1 and H0, while BF_10_ < 1 shows evidence for H0: anecdotal evidence (1/3 < BF_10_ < 1), substantial evidence for H0 (1/10 < BF_10_ < 1/3), and strong evidence for H0 (BF_10_ < 1/10).

Lastly, we examined whether the correct guessing of the stimulation condition led to better memory performance following the real relative to the sham tDCS. We used the *F*-test to assess whether correctly guessed stimulation conditions were associated with higher gain from active tDCS. If subjective beliefs about the stimulation had supported the stimulation effects, a higher stimulation gain would be observed alongside accurate guessing. To assess that, within each experiment, we calculated difference scores between active stimulation and the sham for each of the memory outcomes and transformed obtained differences into z-scores to make them comparable across experiments. Rescaled differences from all experiments were then combined into a single dataset (total of 250 observations). This enabled us to achieve high enough power to detect a small-to-medium effect of sham-guessing on the stimulation gain. Namely, G*Power [53] sensitivity analysis showed the ability of the ANOVA model to detect an effect size (f) of 0.22 (equivalent to η^2^ = 0.048) with the power of 0.95 for the *n* = 250 across 2 groups (correctly vs. incorrectly guessed sham). In essence, this approach has the same aim, i.e., to detect any potential placebo-like effect, but regardless of the stimulation site and outcome measure. The main difference is that combining all data into a single dataset enables detecting smaller effect sizes due to higher statistical power.

## 3. Results

### 3.1. Sucsessfullness of Blinding

Table 2 shows data for the successfulness of blinding in each experiment, suggesting that the sham guesses were at the chance level for Experiments 1, 2, and 4. Participants in Experiment 3 had a somewhat higher guessing rate than the chance level, but this was still below statistical significance.

### 3.2. tDCS Effects on Memory

As previously reported [40,41], facilitatory effects of anodal PPC tDCS on AM were observed in Experiments 1 and 2. Furthermore, the verbal and spatial WM performance improvement was identified following the tDCS over right DLPFC (Experiment 4) and left PPC (Experiment 3), respectively. Additionally, RTs were faster in verbal WM tasks following left and right DLPFC as well as right PPC stimulation. Other comparisons in Experiments 3 and 4 did not show significant tDCS effects. The tDCS effects on memory across all experiments are provided in Appendix A.

### 3.3. The Effects of the Participants’ Subjective Beliefs about the Intervention

The interaction effects from repeated measures frequentist and Bayesian two-way ANOVAs with correct guessing of sham condition as the between-subject factor and the stimulation condition as the repeated measures factor are shown in Table 3. We found no evidence of the interaction between correct sham-guessing and stimulation conditions across all experiments (*p*-values range 0.117–0.985). On the contrary, the Bayesian analysis provided evidence in support of the null hypothesis (i.e., no interaction effect), or equal probability of H1 and H0 for the given dataset (BF_10_ range 0.394–1.017).

**Figure 3:**
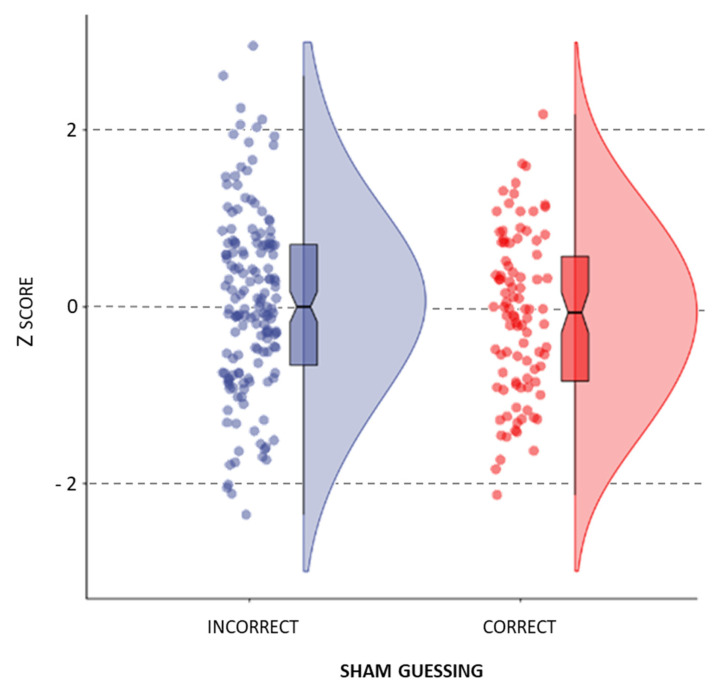
The effect of sham guessing on the standardized anode–sham difference across all observations.

The one-way ANOVA showed no significant effect of correct sham guessing on the anode–sham difference across all observations (*F*_(1,248)_ = 1.607, *p* = 0.206, η_p_ ^2^ = 0.006, BF_10_ = 0.305). That is, there is substantial evidence against the sham guessing effect (evidence towards H0 with 1/10 < BF10 < 1/3), meaning that the ability to correctly guess the sham condition was not associated with a higher gain from active stimulation observed on the behavioral level (Figure 3).

## 4. Discussion

In the present study, we re-evaluated the findings from four crossover, sham-controlled memory experiments to assess the impact of participants’ subjective beliefs about the tDCS type (i.e., real or sham) they received on the obtained results. We found no evidence that participants’ beliefs that they had received the active or sham stimulation impacted their performance in AM and WM tasks. Moreover, we found no evidence for the interaction between the actual stimulation received and correct guessing of the sham protocol. In other words, participants’ assumptions about the intervention did not moderate stimulation effects in any of the experiments. To our knowledge, this is the first study that included multiple experiments to examine the influence of blinding and the possibility of a placebo effect in brain stimulation research on memory.

The analytical approach used in this study sheds light on several important properties of the findings. First, the non-significant interaction effect across all experiments shows that sham guessing does not have a moderating role in a traditional statistical sense. Second, no interaction effect was observed, regardless of whether the main effect of tDCS was observed or not. Furthermore, the Bayesian approach revealed that there is evidence in support of the absence of a sham-guess moderating effect across all experiments; however, this evidence was not convincing for any of the experiments separately. Only the combined data across all observations provided substantial evidence for the absence of placebo-like effects of sham tDCS. Specifically, it is three times more likely that correct sham guessing does not lead to better memory performance following active tDCS (i.e., induce placebo-like effects) than that it does.

Our findings challenge the notion that subjective beliefs about the stimulation type received can impact the results of tDCS studies shown in some previous research [30,37,38,39,54,55]. One of the plausible reasons for this may be the nature of outcome measures examined in each study, i.e., whether subjective or objective outcome measures were used. Namely, there is evidence that the placebo-like effect is particularly evident when it comes to subjective self-report measures, such as participants’ estimation of their own performance [56,57]. For instance, in a study by van Elk et al. [56], even though participants reported enhanced subjective performance from sham tDCS in the cognitive control task, this did not affect objective neurocognitive measures. Other studies which reported the placebo-like effect of tDCS investigated rather subjectively rated or self-reported measures, such as mind-wandering [30], food-craving [39], or depression [54,58]. In comparison with these measures, computerized memory tasks provide more objective assessment. Hence, this type of measure may be less sensitive to the placebo effect and thereby not easily affected by subjective beliefs and expectations about the intervention. Results such as those presented here have been reported for motor and cognitive performance measured with a dexterity task [59], as well as motor learning and neurophysiological responses to placebo transcranial magnetic stimulation [60]. Task measures of memory may act likewise when it comes to placebo effects.

In addition, previous research that examined the placebo effect in tDCS research often involved prior manipulation of participants’ expectations about this intervention [38,56,59]. For instance, researchers provided participants with information sheets that described supposed positive or negative outcomes they can expect from tDCS. Conversely, in our experiments, participants were informed about the aim(s) of the study(ies) (i.e., examining the effects of tDCS on memory functions) as a part of the informed consent, but no special focus was set on their expectations, as the primary goal of the experiments was to examine the effects of the stimulation. However, this does not mean that the participants did not form any expectations about tDCS and its effects. Rabipour et al. [38,59] demonstrated that participants in tDCS studies have variable assumptions and knowledge about the intervention before the experiment—some participants see tDCS as a promising intervention, while others can be skeptical. The relevance of participants’ expectations for tDCS effects has recently been tackled in a narrative review by Braga et al. [36], who argued for a systematic evaluation of participants’ expectations in NIBS research to control their potential confounding effect.

This study tackled the issue of proper blinding of participants, which is crucial for an adequate tDCS experimental design. Blinding has been a matter of debate in recent papers that question the traditional fade-in/out sham protocol as an effective way of keeping participants unaware of the stimulation conditions they receive [31,33,61]. It has been argued that if participants are aware of the stimulation condition they are receiving, the placebo-like effects are more pronounced, compromising the research results. Experiments with a repeated measures design can be more sensitive to unblinding since participants go through at least two sessions which they can compare along at least two dimensions—sensations they felt during stimulation and their subjective level of performance. Nonetheless, the end-of-study guess in this study showed that distinguishing between active and sham protocols was at the chance level for all but one experiment. Therefore, it can be argued that successful blinding could to some extent account for the lack of a placebo effect of tDCS in our experiments. Of note, even in the experiment with the least successful blinding, no evidence of moderating effects of sham-guessing was found. This implies that even when the blinding is not ideal, the stimulation effects are not compromised, at least for objective outcome measures.

Inconsistent success of blinding across different tDCS studies may be explained by the fact that there is no uniformity in sham protocols applied in tDCS research [35]. A recent meta-analysis of the sham effect in tDCS trials for depression revealed that a sham protocol which consists of ramping the current up and down at the beginning and the end of the sham condition resulted in the lowest placebo response (i.e., reduction of symptoms as a result of sham stimulation) [58]. Since our experiments also employed the double ramp-up/down sham protocol, we provide additional evidence in favor of its use for successful blinding and avoiding a placebo response.

Disparities in sham protocols are not the only possible source of inconsistent blinding—tDCS studies are highly heterogeneous regarding applied current intensity as well as electrode sizes and electrode placements, which can cause various cutaneous sensations and interfere with blinding. For instance, we observed higher guessing success in Experiment 3, which employed a somewhat higher current intensity than in Experiments 1 and 2. In general, past studies have shown that participants can detect more nuanced differences in sensations in studies that employ higher current intensities [32,62], smaller electrode sizes [27], and have a reference electrode placed over more sensitive areas, such as the supraorbital region [28]. To improve blinding success, different solutions have been proposed, such as developing novel “active” sham protocols that would diminish differences in scalp sensations between real and sham stimulations [63] or applying topical anesthetics on the skin to control the side effects [64]. Overall, blinding has been identified as an essential component for successful tDCS experimental designs and further improvements of the blinding procedure have been recommended. However, the results reported here question the necessity of these additional efforts to ensure blinding, at least when memory tasks are used as the outcome measures.

When it comes to blinding estimation, it is questionable whether the end-of-study guess appropriately reflects the experiences the participants had during the sessions considering the factors such as forgetting and the forced-choice nature of the measure. It could be argued that the validity of the end-of-study guess depends on one’s ability to accurately remember previous stimulation sessions. Namely, when asked to guess the sham condition, people may rely on their memory of the sensations they experienced in each session (e.g., one could associate real tDCS with the session in which a higher intensity of sensations was experienced), or they could try to guess based on the perceived level of the task performance (e.g., one could believe that the session in which they had better performance must have been the real tDCS). In both cases, the end-of-study guess could be criticized as a valid measure of blinding because the judgement depends on one’s ability to recall past experiences. However, our findings provide no support for this view, since we did not find sham-guessing to be less successful on average in experiments that had a longer time gap between guessing and the first session (Experiments 3 and 4) in comparison to those with the shorter time gap (Experiments 1 and 2).

Some authors advocate for a more comprehensive blinding evaluation which would include asking the participants if they think the stimulation is on while receiving it and to estimate how confident they are in their answer [28,32,34,61]. However, this may cause participants to overly focus on side-effects throughout the session, which can itself compromise blinding as well as the outcome measures of interest. Another alternative for blinding estimation would be to ask the participants to guess the stimulation condition immediately after the session and to include an evaluation of confidence in their assessment. The benefit of this approach is that it evaluates both blinding and the placebo effect in tDCS studies more systematically and comprehensively. Still, this approach can be criticized because the answers might be confounded by one’s readiness to revert previous choices (e.g., if the first session is perceived as real, people will differ in the readiness to label the second session as real too). More importantly, the downside of this approach may be that it prompts participants to think more about the type of stimulation they are receiving than they would normally do.

Thus, the most efficient way to minimize the effects of beliefs about the stimulation type could be to put less emphasis on the fact that there is a real and sham stimulation and direct participants’ attention elsewhere. Namely, to avoid participants overfocusing on cutaneous sensations and the type of stimulation they are receiving (sham or real), it is recommended to include activities or tasks during the stimulation. For example, in our experiments, participants performed different cognitive tasks that occupied their attention during the stimulation. This kind of cognitive engagement during stimulation, besides having the potential to promote after-effects of stimulation [65], can distract participants from stimulation-induced sensations and make them less able to contrast experiences during real and sham sessions.

Finally, several limitations of the current study need to be highlighted and addressed in future research. First, the experiments presented here were not specifically designed to assess the placebo-like effects of tDCS but were focused on assessing the potential facilitatory effect of tDCS on AM and WM. Therefore, simple sham-guess was used to assess the successfulness of blinding as a proxy of one’s awareness of the stimulation condition. To expand on these findings and untangle the differences between blinding and awareness, the future studies may include additional measures of awareness, such as confidence ratings [32] (e.g., How sure are you that this was the sham/real stimulation?) and analyze it in relation to the tDCS effect. This could also enable better distinction between placebo and chance-based guessing. Furthermore, as there are other factors that can moderate tDCS effects [42,66,67], the future studies may explore the potential interaction between successfulness of blinding and variables such as tDCS-induced sensations, mood/affective state of the participants, task difficulty, task modality, stimulation parameters, repeated vs. single tDCS session, etc. Finally, it is important to note that these findings should not be interpreted as if proper blinding in tDCS studies is irrelevant. On the contrary, all measures to assure adequate blinding should be put in place when conducting the tDCS experiments, and the successfulness of blinding should be evaluated and consistently reported. However, when blinding is compromised, the results should not be dismissed before statistically assessing the effect of the awareness of the stimulation condition on the cognitive outcomes.

## 5. Conclusions

This study demonstrated that placebo-like effects stemming from participants’ beliefs about the stimulation type they received are unlikely to influence the results in the research of memory neuromodulation with tDCS in healthy participants, at least when computerized AM and WM tasks with automatic scoring are used as outcome measures.

## Figures and Tables

**Table 1:** Main methods’ characteristics of the Experiments 1–4.

Study	Experiment	tDCS ^1^	Memory Outcome
Bjekić et al. (2019) [40]	Experiment 1 (*N* = 20, 11 females, mean ± *SD* age 26.40 ± 3.71)	Offline protocol Electrode montage: left PPC-contralateral cheek Current intensity: 1.5 mA	Associative memory Task: Face–word memory task Outcome measures: The number of correctly recalled face–word pairs
	Experiment 2 (*N* = 21, 12 females, mean ± *SD* age 24.15 ± 2.74)	Offline protocol Electrode montage: right PPC-contralateral cheek Current intensity: 1.5 mA	Associative memory Task: Object–location memory task Outcome measures: The number of correctly recalled object–location pairs
Živanović et al. (2021) [41] ^2^	Experiment 3 (*N* = 21, 12 females, mean ± *SD* age 26.76 ± 4.83)	Offline protocol Electrode montage: left DLPFC-contralateral cheek left PPC-contralateral cheek Current intensity: 1.8 mA	Working memory Tasks: Spatial and verbal 3-back task Outcome measures: The percentage of hits and the reaction time (RT)
	Experiment 4 (*N* = 21, 12 females, mean ± *SD* age 26.43 ± 4.78 years)	Offline protocol Electrode montage: right DLPFC-contralateral cheek right PPC-contralateral cheek Current intensity: 1.8 mA	Working memory Tasks: Spatial and verbal 3-back task Outcome measures: The percentage of hits and the reaction time (RT)

^1^ In Experiments 1 and 2, the stimulation was delivered via Jonos-4 (Electronic Design Medical D.O.O., Belgrade, Serbia), while in Experiments 3 and 4, STMISOLA (BIOPAC Systems Inc., Goleta, CA, USA) was used. ^2^ Živanović et al. [41] report on one more experiment that examined the online effects of left PPC/DLPFC tDCS on WM. Here, we decided to focus on the influence of sham guesses in offline tDCS protocols and therefore the online experiment is omitted. Note: tDCS—Transcranial Direct Current Stimulation; PPC—Posterior Parietal Cortex; DLPFC—Dorsolateral prefrontal cortex; WM—Working Memory.

**Table 2:** The proportions of participants who accurately guessed the sham condition and the results of the chi-square test.

Study	Correct Sham Guesses	Expected Sham Guesses ^1^	χ^2^	*p*
Experiment 1	7/20 (35%)	10/20 (50%)	1.800	0.180
Experiment 2	8/21 (38%)	10.5/21 (50%)	1.190	0.275
Experiment 3	11/21 (52%)	7/21 (33%)	3.429	0.064
Experiment 4	5/21 (24%)	7/21 (33%)	0.857	0.355

^1^ For Experiments 1 and 2, the expected proportion of guesses was 1:1 (i.e., 0.50); For Experiments 3 and 4, the expected proportion of guesses was 1:2 (i.e., 0.33).

**Table 3:** The interaction effects between correct intervention guessing (i.e., sham guessed correctly or not) and stimulation condition (real vs. sham) for the outcome measures (accuracy and RTs).

Study ^1^	Locus	Memory Task	Accuracy				RT			
			*F*	*p*	η_p_^2^	BF_10_	*F*	*p*	η_p_^2^	BF_10_
Experiment 1	Left PPC	Face–word	0.057	0.814	0.003	0.408				
Experiment 2	Right PPC	Object–location	1.244	0.279	0.061	0.588				
Experiment 3	Left DLPFC	Verbal 3-back	0.850	0.368	0.043	0.553	0.077	0.785	0.004	0.410
		Spatial 3-back	0.000	0.985	0.000	0.394	2.701	0.117	0.124	1.017
	Left PPC	Verbal 3-back	1.250	0.277	0.062	0.575	0.154	0.699	0.008	0.396
		Spatial 3-back	0.011	0.916	0.001	0.402	0.022	0.883	0.001	0.414
Experiment 4	Right DLPFC	Verbal 3-back	0.031	0.863	0.002	0.443	0.382	0.544	0.020	0.462
		Spatial 3-back	0.444	0.513	0.023	0.508	1.220	0.283	0.060	0.574
	Right PPC	Verbal 3-back	1.622	0.218	0.079	0.553	0.017	0.897	0.001	0.433
		Spatial 3-back	1.239	0.279	0.061	0.717	0.602	0.447	0.031	0.476

^1^ The *F* statistic and exact *p*-values are presented for the interaction effects, alongside partial eta squared as a measure of effect size and BF_10_ as a measure of relative H1/H0 odds. The main effect of sham-guess in this ANOVA model is presented in Appendix A.

## Data Availability

Data used in this study are available for re-analysis and validation upon request to the corresponding author.

## Appendix A

The main effects of tDCS on memory are already reported by Bjekić et al. [40] and Živanović et al. [41]. Here, we summarize the results of using the Bayesian approach alongside the same analyses as reported in the studies. Namely, Bayesian and frequentist repeated measures analyses of variance (ANOVA) were performed on the dataset, with the stimulation condition as a within-subject factor, comparing active tDCS conditions to sham (anodal PPC vs. sham in Experiments 1 and 2; anodal PPC vs. sham and anodal DLPFC vs. sham in Experiments 3 and 4).

**Table A1:** tDCS effects on memory performance (Experiments 1–4).

Study	Comparison	Memory Task	Accuracy ^1^			RT ^1^				
			*F*	*p*	η_p_^2^	BF_10_	*F*	*p*	η_p_^2^	BF_10_
Experiment 1	Left PPC vs. sham	Face–word	5.446	0.031	0.223	2.419				
Experiment 2	Right PPC vs. sham	Object–location	4.516	0.046	0.184	2.565				
Experiment 3	Left DLPFC vs. sham	Verbal 3-back	0.110	0.744	0.005	0.314	5.355	0.031	0.211	2.039
		Spatial 3-back	0.355	0.558	0.017	0.343	0.001	0.975	0.000	0.304
	Left PPC vs. sham	Verbal 3-back	2.872	0.106	0.126	0.901	2.275	0.147	0.102	0.703
		Spatial 3-back	6.176	0.022	0.236	2.540	0.103	0.715	0.005	0.313
Experiment 4	Right DLPFC vs. sham	Verbal 3-back	7.179	0.014	0.264	3.484	7.856	0.011	0.282	4.680
		Spatial 3-back	3.354	0.082	0.144	1.076	2.817	0.109	0.123	0.924
	Right PPC vs. sham	Verbal 3-back	3.924	0.062	0.164	1.241	6.252	0.021	0.238	2.667
		Spatial 3-back	1.526	0.231	0.071	0.551	1.697	0.207	0.078	0.582

^1^ Significant effects (*p* < 0.05) are marked in bold. For Experiments 1 and 2, the analyses were run on the average AM scores from the first and second learning blocks; while for Experiments 3 and 4, we used the number of hits and reaction time as outcome measures. As in the original analyses, the WM hits scores were centered on the order of the session to control for practice effects.

One could also expect that those who correctly guessed the sham condition had superior memory performance; that is, a significant main effect of the between-subject factor sham guess in the mixed ANOVA model. However, no main effects of guessing were found for any of the outcome measures (Table A2), except for face–word AM.

**Table A2:** The main effects of the sham guess factor on accuracy and RTs in mixed ANOVA (Experiments 1–4).

Study	Locus	Memory Task	Accuracy ^1^			RT ^1^				
			*F*	*p*	η_p_^2^	BF_10_	*F*	*p*	η_p_^2^	BF_10_
Experiment 1	Left PPC	Face–word	5.289	0.034	0.227	1.864				
Experiment 2	Right PPC	Object–location	2.971	0.101	0.135	1.191				
Experiment 3	Left DLPFC	Verbal 3-back	0.641	0.433	0.033	0.592	0.145	0.708	0.008	0.605
		Spatial 3-back	0.251	0.622	0.013	0.632	0.754	0.396	0.038	0.604
	Left PPC	Verbal 3-back	0.002	0.962	0.000	0.552	0.172	0.683	0.009	0.534
		Spatial 3-back	0.266	0.612	0.014	0.630	0.000	0.984	0.000	0.462
Experiment 4	Right DLPFC	Verbal 3-back	0.610	0.444	0.031	0.615	0.125	0.728	0.007	0.509
		Spatial 3-back	0.914	0.351	0.046	0.618	0.121	0.732	0.006	0.482
	Right PPC	Verbal 3-back	0.145	0.707	0.008	0.521	0.000	0.995	0.000	0.515
		Spatial 3-back	0.600	0.448	0.031	0.565	0.005	0.945	0.000	0.510

^1^ Significant effects (*p* < 0.05) are marked in bold.
